# Preoperative electromagnetic navigation bronchoscopy‐guided one‐stage multiple‐dye localization for resection of subsolid nodules: A single‐center pilot study

**DOI:** 10.1111/1759-7714.14283

**Published:** 2021-12-23

**Authors:** Jong Hwan Jeong, Hyeongbin Park, Chang‐Min Choi, Ju Hyun Oh, Geun Dong Lee, Dong Kwan Kim, Hee Sang Hwang, Se Jin Jang, Sang Young Oh, Mi Young Kim, Wonjun Ji

**Affiliations:** ^1^ Department of Pulmonary and Critical Care Medicine, Asan Medical Center University of Ulsan College of Medicine Seoul South Korea; ^2^ Division of Thoracic and Cardiovascular Surgery, Department of Internal Medicine Ewha woman's University college of medicine Seoul South Korea; ^3^ Department of Pathology Asan Medical Center Seoul South Korea; ^4^ Department of Radiology, Asan Medical Center University of Ulsan College of Medicine Seoul South Korea

**Keywords:** dye localization, electromagnetic navigation bronchoscopy, multiple subsolid pulmonary nodules

## Abstract

**Background:**

Electromagnetic navigation bronchoscopy (ENB)‐guided transbronchial dye marking and video‐assisted thoracoscopic surgery (VATS) is an emerging technique that enables successful resection of multiple small subsolid pulmonary nodules. The aim of this study was to evaluate the accuracy and safety of preoperative ENB‐guided transbronchial multiple dye localization for VATS resection of subsolid pulmonary nodules.

**Methods:**

As a single‐center pilot study, we recruited patients with at least two small or subsolid pulmonary nodules. Multiple‐dye localization was performed by intraoperative ENB‐guided transbronchial injection of an indigo carmine dye. The patients underwent VATS for sublobar resection immediately after localization. The accuracy of ENB‐guided dye marking was checked.

**Results:**

ENB‐guided one‐stage multiple dye localization was conducted for 18 pulmonary nodules in seven patients between September 2018 and December 2019. The mean diameter of the pulmonary nodules was 9.3 mm (range, 4–18) and the mean distance from the pleura to pulmonary nodule was 6 mm (range, 1–17 mm). ENB‐guided transbronchial multiple dye localization was successfully performed in 94.4% (17/18), and the accuracy of ENB‐guided dye marking was 88.2% (15/17). When two nodules were not seen in intraoperative fields, anatomical sublobar resection was performed. There was no conversion to thoracotomy and operative mortalities. Among the seven patients, only one patient showed mild intrabronchial bleeding but stopped spontaneously. The changes in lung function after multiple wedge resections (−1.6% to 24.8%) were tolerable level.

**Conclusions:**

ENB‐guided one‐stage transbronchial dye localization showed accurate and safe intraoperative identification of multiple subsolid pulmonary nodules. A large scale prospective clinical study is warranted.

## Graphical abstract

We performed ENB‐guided one‐stage transbronchial dye localizations for multiple lung nodules and investigated follow‐up results regarding changes in lung function after resection. Safe and successful outcomes were obtained and the postoperative decrease in lung function due to multiple pulmonary nodules was tolerable.

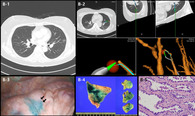

## INTRODUCTION

The incidence of lung nodules is rising due to the increasing tendency of screening for lung cancer using chest computed tomography (CT). Two large prospective randomized controlled trials, the National Lung Screening Trial (NLST) and NELSON study, showed that early detection of lung cancer by low dose CT (LDCT) screening leads to a significant reduction in mortality from lung cancer and a significant shift in early‐stage malignancies at the time of diagnosis.^1^, ^2^ Thus, there is a growing need for appropriate surgical resection of early‐stage lung cancers detected upon screening. Moreover, unlike in the past, adenocarcinoma has been reported to be the most frequent histopathological type of lung cancer.^3^, ^4^ It commonly presents with a subsolid shape on CT scans and sometimes manifests as a multifocal pattern.^5^, ^6^, ^7^

Several prior studies showed that video‐assisted thoracoscopic surgery (VATS) biopsy for small subsolid nodules is challenging due to their impalpability.^8^, ^9^ Therefore, several techniques have been implemented to localize the subsolid nodules in the operating room. The most widely used technique is CT‐guided transthoracic localization with hook wire. However, the technique has a limitation in that localization of multiple lesions is impractical due to the associated complications, such as pneumothorax or hemothorax.^10^ Therefore, there is a need for techniques that allow preoperative localization at multiple sites in patients with multiple small or subsolid nodules suspected of early lung cancer.

Nowadays, Electromagnetic navigation bronchoscopy (ENB) is an emerging bronchoscopic technology.^11^ It converts the CT image of a tracheobronchial airway tree into a three‐dimensional virtual map and uses an electromagnetic tracking system to guide a steerable probe to approach peripheral lung lesions.^12^ According to previous studies, promising outcomes have been reported in terms of the accuracy and safety of ENB‐guided transbronchial needle aspiration or biopsy.^13^, ^14^ It has also been applied as a useful preoperative localization tool for precise and successful VATS resection by transbronchial dye marking to target lung lesions these days.^8^, ^15^ Furthermore, this technique enables injection of dye to multiple target lesions for preoperative localization with a single procedure; it confers the advantage of allowing the simultaneous surgical resection on multiple lung nodules with a one‐stage operation. Nonetheless, most previous studies have reported results for patients with only a single lung nodule, and data on the simultaneous resection of multiple lung nodules using ENB‐guided localization are scarce.^13^, ^15^

In this study, we aimed to validate the accuracy and safety of preoperative ENB‐guided transbronchial dye localization for VATS resection of multiple subsolid pulmonary nodules suspected of lung cancer. Furthermore, we also investigated follow‐up results regarding changes in lung function after resections of multiple lung nodules.

## METHODS

### Study population

This study was a retrospective, single‐center pilot study on patients aged 18–80 years, who could medically tolerate general anesthesia. We selected patients who had two or more subsolid pulmonary nodules in the ipsilateral lung, and at least one of the nodules was highly suspected of early malignancy. The exclusion criteria in this study were patients who were intolerant of general anesthesia, who had severe cardiopulmonary disease, pregnant women, and patients who did not consent to the study. All participants provided their written informed consent. The Institutional Review Board of the Asan Medical Center approved this study (approval no. 2020‐1009).

### Preoperative electromagnetic navigation bronchoscopy‐guided one‐stage multiple‐dye localization

The enrolled patients underwent an enhanced chest CT, following the inspiration/expiration protocol, according to the recommendation of the manufacturer (Veran Medical Technologics) to reconstruct a 3D bronchoscopy map before resection. One physician planned preoperative bronchoscopy with the SPiN system and SPiN planning software (Veran Medical Technologics). Primarily, we tried to directly target the nodules. However, if direct access to the target nodules was not possible, such as when they were far from the pleural surface or without a bronchus sign, we performed dye marking to the pleura, which was the nearest site from the target lesions.

**FIGURE 1:**
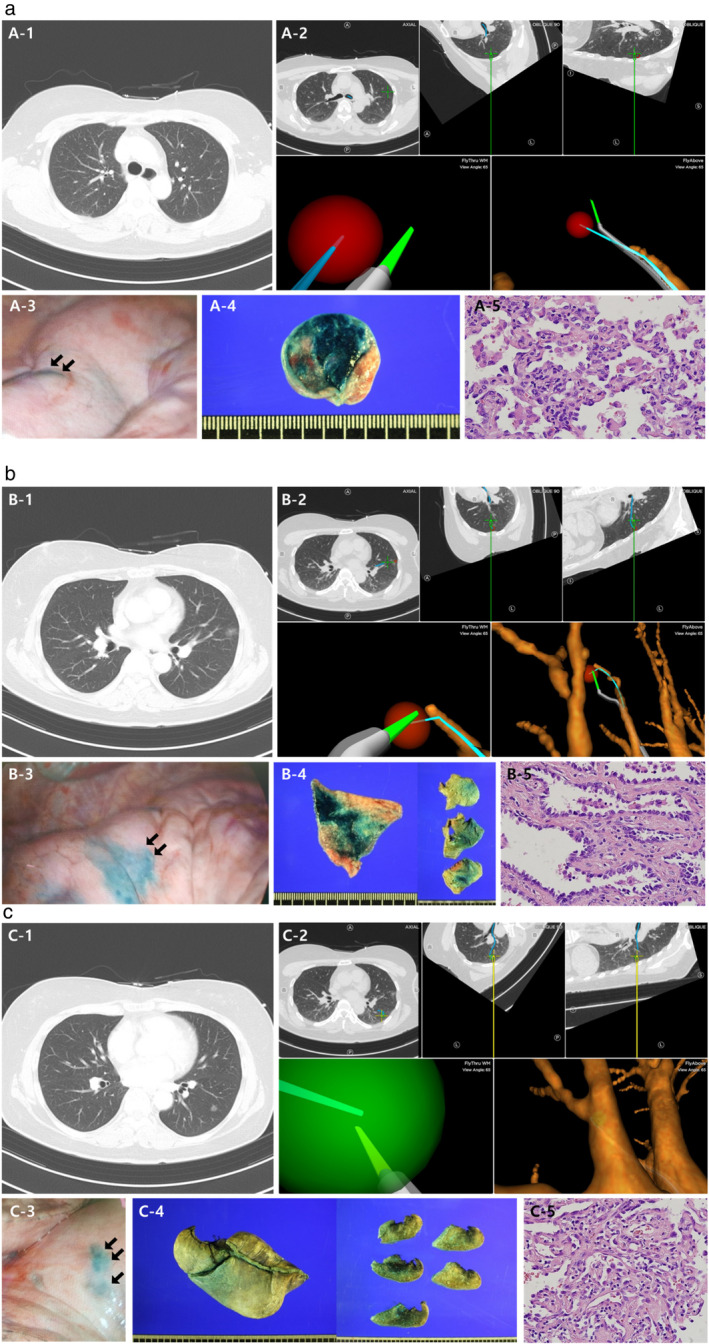
ENB‐guided one‐stage multiple dye localization case. (a) A ground‐glass opacity (GGO) nodule in the anterior segment of the LUL. (b) A GGO nodule in the lingular division of the LUL. (c) A GGO nodule of LLL superior segment; (a, b, c‐1) chest CT image; (a, b, c‐2) the navigation screen during the transbronchial approaches; (a, b, c‐3) the lung surface in intraoperative fields after transbronchial dye injection using indigo carmine; (a, b, c‐4) the resected specimen stained by indigo carmine; (a, b, c‐5) the photomicrographs of the resected tissue (X400)

The patients underwent general anesthesia and ENB‐guided dye marking was performed just before surgical incision in the operating room. Indigo carmine (1 ml) was injected into each target using a working catheter under an electromagnetic guide in a one‐stage method; subsequently, 0.9% normal saline (1 ml) was injected into the lumen of the bronchoscopy and in the needle for dye flushing. After sufficient localization, VATS for sublobar resection was immediately conducted for surgical removal of lung nodules and intraoperative evaluation of dye marking (Figure 1). All subjects underwent baseline pulmonary function tests before surgery and postoperative pulmonary function tests 6–12 months after surgery.

### Measurement of accuracy and safety of ENB guide dye localization

The primary endpoint in this study was the accuracy of preoperative ENB‐guided dye localization. We defined whether the dye localizations were visible or distinguishable on lung surface in intraoperative fields. The secondary endpoints were success rate to reach the target lesions using ENB‐guided technique, the safety profile of ENB‐guided dye localization, successful resection rate of lung nodules, the extent of resection, pathologic findings of resected lung nodules, and changes in postoperative pulmonary functions compared with baseline values.

### Statistical analysis

Data obtained from the participants were used to compare the demographic and baseline values among patients. Descriptive analyses were performed for the primary and secondary endpoints; for the analysis of the difference between a group of successful operations and failed operations, the *t*‐test was conducted for continuous variables and Pearson's chi‐square test for categorical variables. All statistical analyses results were considered significant if the *p*‐value was less than 0.05. Statistical analyses were performed using IBM SPSS version 26.0 (IBM Corporation).

## RESULTS

### Baseline characteristics of the patients and nodules

Between September 2018 and December 2019, a total of seven patients with 18 lesions underwent ENB‐guided one‐stage dye localization. The baseline characteristics of the patients and nodules are described in Table 1. The ages of the enrolled subjects ranged from 41 to 66 (median age 53 years), and three subjects (42.8%) were male. Spirometry was performed on all subjects, the average values of forced expiratory volume in the first second and forced volume vital capacity were 90.7% and 89.7%, respectively. The number of lung nodules was three in four patients and two in three patients. The mean diameter of the pulmonary nodules was 9.3 mm (range, 4–18) and the mean distance from the pleura to pulmonary nodule was 6 mm (range, 1–17 mm). Of the 18 lung nodules, five (27.8%) were located in the right lung and 13 (72.2%) were located in the left lung. There were seven nodules (38.9%) located in the upper lobes, and 11 nodules (61.1%) in the lower lobes.

**TABLE 1:** Baseline characteristics of patients with multiple small, subsolid pulmonary nodules

Cases	Age/sex	Comorbidities	Pulmonary function FVC/FEV1/ratio, %)	DLCO (%)	Number of lung nodules	Size of lung nodules (mm)	Location of lung nodules (segment)
Case 1	51/F	Hypothyroidism	86/87/83	82	3	14	LLL posterior
						7	LUL anterior
						6	LLL superior
Case 2	53/F	Papillary thyroid cancer, myoma	92/96/83	86	3	14	Lingular superior
						9	LLL superior
						6	LUL anterior
Case 3	41/M	Ameloblastoma	83/81/77	84	3	10	RLL laterobasal
						7	RLL anterobasal
						4	RLL mediobasal
Case 4	66/F	DM, HTN, endometrial cancer	103/106/77	102	2	13	Lingular superior.
						8	LLL anterobasal
Case 5	44/F	‐	90/98/93	85	3	12	LLL laterobasal
						9	LLL superior
						7	LLL posterobasal
Case 6	59/M	Papillary thyroid cancer	73/77/82	100	2	18	RUL anterior
						6	RLL superior
Case 7	60/M	‐	101/90/68	‐	2	14	LUL apical
						4	LUL anterior

*Abbreviations*: DLCO, diffusing capacity of the lung for carbon monoxide; F, female; FEV1, forced expiratory volume in 1 second; FVC, forced vital capacity; M, male; LLL, left lower lobe; LUL, left upper lobe; RLL, right lower lobe; RUL, right upper lobe.

### Results of electromagnetic navigation bronchoscopy‐guided one‐stage multiple‐dye localization

The results of ENB‐guided one‐stage multiple‐dye localization are described in Table 2. Of the 18 nodules, 13, three, and two nodules were of ground‐glass opacity, solid, and part solid, respectively. We performed localization using indigo carmine in all cases. ENB‐guided transbronchial multiple‐dye localization was successfully performed in 94.4% (17/18) cases, and there was only one case that failed to approach the target lesion, which was located in the superior segment of the left lower lobe. The accuracy of ENB‐guided dye marking was 88.2% (15/17). Two nodules were invisible on lung surface in intraoperative fields; anatomical sublobar resections were performed to resect them. Final diagnosis was adenocarcinoma in 12 (66.7%), ameloblastoma in three (16.7%), and benign in three (16.7%) cases. VATS was performed successfully in all patients (100%). While the initial surgical approaches were wedge resections, surgical extents were extended to lobectomy in four nodules and segmentectomy in three nodules, because the results of the intraoperative frozen biopsy were confirmed as invasive cancer.

**TABLE 2:** Results of electromagnetic navigation bronchoscopy guided one‐stage multiple dye localization

Cases	Location of lung nodules	Nodule characteristics	Type of dye used	Localization results	Extent of resection	Pathological finding
Case 1	LLL posterior	GGO	indigo carmine	Success	Segmentectomy	Invasive ADC
	LUL anterior	GGO		Success	Wedge resection	AIS
	LLL superior	GGO		Inaccessible	Wedge resection	AIS
Case 2	Lingular superior	GGO	indigo carmine	Success	Segmentectomy	Invasive ADC
	LLL superior	GGO		Success	Wedge resection	AIS
	LUL anterior	GGO		Success	Wedge resection	AIS
Case 3	RLL laterobasal	Solid	indigo carmine	Success	Wedge resection	Ameloblastoma
	RLL anterobasal	Solid		invisible	Wedge resection	Ameloblastoma
	RLL mediobasal	Solid		Success	Wedge resection	Ameloblastoma
Case 4	Lingular superior	GGO	indigo carmine	Success	Wedge resection	Focal interstitital fibrosis
	LLL anterobasal	GGO		Success	Wedge resection	Focal interstitital fibrosis
Case 5	LLL laterobasal	GGO	indigo carmine	Success	Lobectomy	Minimally invasive ADC
	LLL superior	GGO		Success	Lobectomy	Invasive ADC
	LLL posterobasal	GGO		invisible	Lobectomy	Minimally invasive ADC
Case 6	RUL anterior	Part solid	indigo carmine	Success	Lobectomy	Invasive ADC
	RLL superior	GGO		Success	Wedge resection	Minimally invasive ADC
Case 7	LUL apical	GGO	indigo carmine + ICG	Success	Wedge resection	AAH
	LUL anterior	Part solid		Success	Segmentectomy	Invasive ADC

*Abbreviations*: AAH, atypical adenomatous hyperplasia; ADC, adenocarcinoma; AIS, adenocarcinoma in situ; GGO, ground‐glass opacity; ICG, indocyanine green; LLL, left lower lobe; LUL, left upper lobe; RLL, right lower lobe; RUL, right upper lobe.

### Safety profile and adverse events of ENB‐guided dye localization

There was no conversion to thoracotomy and operative mortalities. Among the seven patients, there were no major complications, including moderate to severe bleeding or pneumothorax during ENB‐guided dye localization. Only one patient showed mild intrabronchial bleeding, but it stopped spontaneously.

### Changes in postoperative pulmonary function

Table 3 shows the results of the changes between baseline and postoperative pulmonary function. The range of baseline FEV1 was 2.35–3.67 L and the range of postoperative FEV1 was 2.02–3.55 L. FEV1 decreased by 1.6%–24.8% after the surgery and the average loss of FEV1 was 0.30 L (10.7%). The ranges of baseline and postoperative FVC were 2.82–4.75 L and 2.61–4.61 L, respectively.

**TABLE 3:** Pre‐ and postoperative pulmonary function

	Baseline value (% of predicted value)			Postoperative value (% of predicted value)			Change of FEV1, L (% of change)
Cases	FVC, L	FEV1, L	FEV1/FVC	FVC, L	FEV1, L	FEV1/FVC	
Case 1	2.82 (86)	2.35 (87)	83	2.61 (80)	2.02 (75)	77	−0.33 (−14.0)
Case 2	3.34 (92)	2.79 (96)	83	3.16 (89)	2.35 (83)	74	−0.44 (−15.8)
Case 3	4.75 (83)	3.67 (81)	77	4.61 (81)	3.55 (80)	77	−0.12 (−3.3)
Case 4	3.20 (103)	2.47 (106)	77	3.13 (102)	2.43 (105)	78	−0.04 (−1.6)
Case 5	2.99 (90)	2.78 (98)	93	2.35 (71)	2.09 (74)	89	−0.69 (−24.8)
Case 6	3.12 (73)	2.55 (77)	82	2.83 (67)	2.48 (77)	88	−0.07 (−2.7)
Case 7	4.60 (101)	3.12 (90)	68	4.49 (98)	2.70 (78)	60	−0.42 (−13.5)

*Abbreviations*: FEV1, forced expiratory volume in 1 second; FVC, forced vital capacity; L, liter.

## DISCUSSION

In this retrospective pilot study, we successfully performed ENB‐guided one‐stage multiple‐dye localization in 94.4% (17/18) cases, and the accuracy of dye marking was 88.2% (15/17). No major complications or procedure‐related deaths were reported, and only mild intrabronchial bleeding was reported in one case (5.6%). Despite the inclusion of lobectomy or segmentectomy cases, the reductions of postoperative lung functions after multiple wedge resections were at a tolerable level.

The most widely used technique is CT‐guided, hook‐wire localization that was considered successful in 84%–97.6% of cases.^16^, ^17^, ^18^ Other techniques with CT‐guided percutaneous localization, including placement of a microcoil,^19^ lipiodol,^20^ and injection of dye,^21^ had success rates of 93%–100%. Nonetheless, the most common complications of CT‐guided percutaneous marking were pneumothorax and intrapulmonary bleeding, and hemothorax and air embolism occurred as rare complications. Some studies with CT‐guided hook‐wire insertion have described that pneumothorax, intrapulmonary bleeding, and hemothorax were confirmed in 5.9%–38%, 5.9%–27.1%, and 0%–0.6% of cases, respectively.^10^, ^16^, ^22^ Further, the occurrence of systemic air embolism, the most serious complication, has also been reported.^23^ In the present study, we found a satisfactory success rate, which was comparable to that of CT‐guided localization methods. Furthermore, we observed no major complication aside from mild intrabronchial bleeding in only one case. Therefore, our study demonstrates that the ENB‐guided transbronchial localization is as successful as CT‐guided localization techniques and may be considered superior in terms of safety.

Bowling et al. recently reported that ENB‐guided transbronchial dye marking procedure was regarded as sufficient for surgical resection in 91.3% cases in a large scale prospective cohort study.^15^ Cho et al. also used ENB‐guided dye markings for 32 lesions in 25 patients to localize pulmonary nodules and achieved an 87.5% success rate.^24^ Meanwhile, ENB‐guided transbronchial approach has a relatively low incidence rate of procedure‐related complications.^15^, ^24^, ^25^, ^26^ Most studies have reported no specific complications except mild intrabronchial bleeding; one large scale study of 1000 subjects described the occurrence of pneumothorax, bronchopulmonary hemorrhage, and respiratory failure in 4.9, 2.3, and 0.6% cases, respectively.^13^ Nonetheless, most existing studies on ENB‐guided methods were conducted only for one or two pulmonary nodules. In our study, we performed ENB‐guided one‐stage localizations for two or more lung lesions, and confirmed the safe and successful outcomes of the procedure and surgical resection compared to previous reports.

It is generally known that there is a notable decline in FEV1 immediately after lobectomy, but it shows a gradual improvement over 3–6 months afterwards.^27^, ^28^ Previous studies have reported that the mean FEV1 level decreased by 9%–17% following lobectomy in patients with lung cancer.^29^, ^30^, ^31^ The concern is that the resection of multiple lung nodules suspected of lung cancer may cause a substantial loss of lung function. To our knowledge, there is a lack of data on changes in lung function after localization and resection of the lung nodules, whereas, several previous studies have reported on the efficacy or safety of the ENB‐guided localization technique. In the present study, we found that FEV1 decreased by 1.6%–24.8% after surgery, and the mean percentage of reduction in FEV1 was 10.7%. Despite the inclusion of patients who underwent lobectomy or segmentectomy, the changes in lung function following multiple wedge resections were not significant in our study. In particular, two patients with only multiple wedge resections (Cases 3, 4) showed minimal changes of postoperative FEV1.

Long et al.^32^ performed electromagnetic transthoracic nodule localization for 31 nodules in 30 patients. In that study, 94% of the nodules were successfully localized, and failure was reported in only two cases. Although this percutaneous transthoracic approach shows a good localization accuracy, it is associated with procedural‐related complications, such as pneumothorax and bleeding. Meanwhile, the main difference between our study and the research above was in the method of approach to lesions. In our study with transbronchial approaches, there was a limitation that the localization failed in areas that were less accessible using bronchoscopy, such as those located in the superior segment of the left lower lobe (Case 1). Thus, if we apply both transthoracic and transbronchial routes at once, we can expect a better outcome by enabling localization in areas where bronchoscopy is inaccessible and localization of multiple lesions simultaneously with low complication rates.

In the current study, there were three lung lesions in one patient (Case 3) and the pathological results were confirmed as ameloblastoma. Ameloblastoma is an uncommon odontogenic tumor that mainly affects the mandible and maxilla.^33^ Although metastasis of ameloblastoma mainly involves the lung, metastasizing ameloblastomas are extremely rare.^34^, ^35^ In Case 3 in our study, a 43‐year‐old man was first diagnosed with ameloblastoma in 2009 after mandible cyst resection. In 2018, multiple lung nodules suspected of pulmonary metastatic ameloblastoma were identified in chest CT. Video‐assisted thoracoscopic wedge resection was performed through the CT‐guided hook‐wire localization method. Metastasizing ameloblastoma was finally confirmed in the pathology report. Since there was no evidence of metastasis involving another area, a metastasectomy was performed for all resectable pulmonary nodules in a multidisciplinary approach. We decided to choose the ENB‐guided transbronchial method for preoperative localization, considering that CT‐guided percutaneous localization of multiple lung lesions was not possible and might have a high risk of complications; we obtained safe and successful outcomes. In addition, the changes in pulmonary functions after multiple wedge resections were at a tolerable level.

The present study has several limitations. First, because the study was conducted as a single‐center, retrospective study with a small sample size of seven patients, the risk of selection bias cannot be excluded. However, our study is worthwhile in that it is a pilot study showing the clinical usefulness of transbronchial one‐stage multiple‐dye localization using an ENB‐guided technique, paving the way for a large prospective clinical trial, and it shows satisfactory outcomes in safety and accuracy. Second, we mainly used the indigo carmine dye alone, which can sometimes be indistinguishable from pleural pigmentation in the operating field; we did not check the efficacy of different combinations of dye markers, such as indocyanine green (ICG), which can potentially overcome these limitations. In one case (Case 7), we performed dye marking using both indigo carmine and ICG, and it showed better visibility and distinguishability. Therefore, further studies are needed to evaluate the effectiveness of various combinations of dye markers and identify the most efficient method. Third, this analysis could not confirm a survival benefit or effect of preventing the lesions from progressing to invasive cancer depending on whether or not each lung nodules were resected. However, given that most atypical adenomatous hyperplasia (AAH) are likely to progress slowly over the years and have a high probability of developing into invasive cancer, surgical resection of all lesions at the first stage can offer the advantage of avoiding an increased risk of postoperative complications associated with aging or post‐surgical adhesions. In addition, the fact that this study showed a tolerable result of postoperative lung function following multiple wedge resection also supports the idea that simultaneous resection may be a better treatment option. Lastly, this analysis did not provide data on the long‐term prognosis in patients who had a resection of multiple lung nodules. Further research on whether simultaneous resection of all lesions in patients with multiple lung nodules can help to improve the long‐term prognosis and survival rate is required.

In conclusion, ENB‐guided transbronchial one‐stage multiple‐dye localization is an efficient and safe tool for guiding sublobar resection of multiple lung nodules. In addition, the postoperative decrease in lung function due to multiple pulmonary nodules was tolerable. A large scale prospective clinical study using ENB‐guided one‐stage multiple transbronchial dye localization is warranted.

## CONFLICT OF INTEREST

Any authors have no competing interests to declare.

## References

[1] Aberle DR , Adams AM , Berg CD , et al. Reduced lung‐cancer mortality with low‐dose computed tomographic screening. N Engl J Med. 2011;365:395–409. 21714641 10.1056/NEJMoa1102873 PMC4356534

[2] de Koning HJ , van der Aalst CM , de Jong PA , Scholten ET , Nackaerts K , Heuvelmans MA , et al. Reduced lung‐cancer mortality with volume CT screening in a randomized trial. N Engl J Med. 2020;382:503–13. 31995683 10.1056/NEJMoa1911793

[3] Travis WD , Brambilla E , Noguchi M , Nicholson AG , Geisinger KR , Yatabe Y , et al. International association for the study of lung cancer/american thoracic society/european respiratory society international multidisciplinary classification of lung adenocarcinoma. J Thorac Oncol. 2011;6:244–85. 21252716 10.1097/JTO.0b013e318206a221 PMC4513953

[4] Lortet‐Tieulent J , Soerjomataram I , Ferlay J , Rutherford M , Weiderpass E , Bray F . International trends in lung cancer incidence by histological subtype: adenocarcinoma stabilizing in men but still increasing in women. Lung Cancer. 2014;84:13–22. 24524818 10.1016/j.lungcan.2014.01.009

[5] Ye T , Deng L , Wang S , Xiang J , Zhang Y , Hu H , et al. Lung adenocarcinomas manifesting as radiological part‐solid nodules define a special clinical subtype. J Thorac Oncol. 2019;14:617–27. 30659988 10.1016/j.jtho.2018.12.030

[6] Chang YL , Wu CT , Lee YC . Surgical treatment of synchronous multiple primary lung cancers: experience of 92 patients. J Thorac Cardiovasc Surg. 2007;134:630–7. 17723810 10.1016/j.jtcvs.2007.06.001

[7] Kim TJ , Goo JM , Lee KW , Park CM , Lee HJ . Clinical, pathological and thin‐section CT features of persistent multiple ground‐glass opacity nodules: comparison with solitary ground‐glass opacity nodule. Lung Cancer. 2009;64:171–8. 18799230 10.1016/j.lungcan.2008.08.002

[8] Awais O , Reidy MR , Mehta K , Bianco V , Gooding WE , Schuchert MJ , et al. Electromagnetic navigation bronchoscopy‐guided dye marking for Thoracoscopic resection of pulmonary nodules. Ann Thorac Surg. 2016;102:223–9. 27157054 10.1016/j.athoracsur.2016.02.040 PMC13296369

[9] Suzuki K , Nagai K , Yoshida J , Ohmatsu H , Takahashi K , Nishimura M , et al. Video‐assisted thoracoscopic surgery for small indeterminate pulmonary nodules: indications for preoperative marking. Chest. 1999;115:563–8. 10027460 10.1378/chest.115.2.563

[10] Hanauer M , Perentes JY , Krueger T , Ris HB , Bize P , Schmidt S , et al. Pre‐operative localization of solitary pulmonary nodules with computed tomography‐guided hook wire: report of 181 patients. J Cardiothorac Surg. 2016;11:5. 26772183 10.1186/s13019-016-0404-4 PMC4715360

[11] Leong S , Ju H , Marshall H , Bowman R , Yang I , Ree AM , et al. Electromagnetic navigation bronchoscopy: a descriptive analysis. J Thorac Dis. 2012;4:173–85. 22833823 10.3978/j.issn.2072-1439.2012.03.08 PMC3378214

[12] Gildea TR , Mazzone PJ , Karnak D , Meziane M , Mehta AC . Electromagnetic navigation diagnostic bronchoscopy: a prospective study. Am J Respir Crit Care Med. 2006;174:982–9. 16873767 10.1164/rccm.200603-344OC PMC2648102

[13] Khandhar SJ , Bowling MR , Flandes J , et al. Electromagnetic navigation bronchoscopy to access lung lesions in 1,000 subjects: first results of the prospective, multicenter NAVIGATE study. BMC Pulm Med. 2017;17:59. 28399830 10.1186/s12890-017-0403-9 PMC5387322

[14] Loo FL , Halligan AM , Port JL , Hoda RS . The emerging technique of electromagnetic navigation bronchoscopy‐guided fine‐needle aspiration of peripheral lung lesions: promising results in 50 lesions. Cancer Cytopathol. 2014;122:191–9. 24323803 10.1002/cncy.21373

[15] Bowling MR , Folch EE , Khandhar SJ , Arenberg DA , Awais O , Minnich DJ , et al. Pleural dye marking of lung nodules by electromagnetic navigation bronchoscopy. Clin Respir J. 2019;13:700–7. 31424623 10.1111/crj.13077

[16] Dendo S , Kanazawa S , Ando A , Hyodo T , Kouno Y , Yasui K , et al. Preoperative localization of small pulmonary lesions with a short hook wire and suture system: experience with 168 procedures. Radiology. 2002;225:511–8. 12409589 10.1148/radiol.2252011025

[17] Chen YR , Yeow KM , Lee JY , Su IH , Chu SY , Lee CH , et al. CT‐guided hook wire localization of subpleural lung lesions for video‐assisted thoracoscopic surgery (VATS). J Formos Med Assoc. 2007;106:911–8. 18063512 10.1016/S0929-6646(08)60061-3

[18] Gonfiotti A , Davini F , Vaggelli L , de Francisci A , Caldarella A , Gigli PM , et al. Thoracoscopic localization techniques for patients with solitary pulmonary nodule: hookwire versus radio‐guided surgery. Eur J Cardiothorac Surg. 2007;32:843–7. 17913505 10.1016/j.ejcts.2007.09.002

[19] Finley RJ , Mayo JR , Grant K , Clifton JC , English J , Leo J , et al. Preoperative computed tomography‐guided microcoil localization of small peripheral pulmonary nodules: a prospective randomized controlled trial. J Thorac Cardiovasc Surg. 2015;149:26–31. 25293355 10.1016/j.jtcvs.2014.08.055

[20] Ikeda K , Nomori H , Mori T , Kobayashi H , Iwatani K , Yoshimoto K , et al. Impalpable pulmonary nodules with ground‐glass opacity: success for making pathologic sections with preoperative marking by lipiodol. Chest. 2007;131:502–6. 17296654 10.1378/chest.06-1882

[21] Lenglinger FX , Schwarz CD , Artmann W . Localization of pulmonary nodules before thoracoscopic surgery: value of percutaneous staining with methylene blue. AJR Am J Roentgenol. 1994;163:297–300. 7518642 10.2214/ajr.163.2.7518642

[22] Huang HZ , Wang GZ , Xu LC , Li GD , Wang Y , Wang YH , et al. CT‐guided Hookwire localization before video‐assisted thoracoscopic surgery for solitary ground‐glass opacity dominant pulmonary nodules: radiologic‐pathologic analysis. Oncotarget. 2017;8:108118–29. 29296228 10.18632/oncotarget.22551 PMC5746130

[23] Ichinose J , Kohno T , Fujimori S , Harano T , Suzuki S . Efficacy and complications of computed tomography‐guided hook wire localization. Ann Thorac Surg. 2013;96:1203–8. 23895891 10.1016/j.athoracsur.2013.05.026

[24] Cho HJ , Roknuggaman M , Han WS , Kang SK , Kang MW . Electromagnetic navigation bronchoscopy‐Chungnam National University Hospital experience. J Thorac Dis. 2018;10:S717–S24. 29732192 10.21037/jtd.2018.03.130 PMC5911745

[25] Muñoz‐Largacha JA , Ebright MI , Litle VR , Fernando HC . Electromagnetic navigational bronchoscopy with dye marking for identification of small peripheral lung nodules during minimally invasive surgical resection. J Thorac Dis. 2017;9:802–8. 28449489 10.21037/jtd.2017.03.18 PMC5394035

[26] Hsu PK , Chuang LC , Wu YC . Electromagnetic navigation‐guided preoperative localization of small malignant pulmonary tumors. Ann Thorac Surg. 2020;109:1566–73. 32032573 10.1016/j.athoracsur.2019.12.037

[27] Brunelli A , Refai M , Salati M , Xiumé F , Sabbatini A . Predicted versus observed FEV1 and DLCO after major lung resection: a prospective evaluation at different postoperative periods. Ann Thorac Surg. 2007;83:1134–9. 17307474 10.1016/j.athoracsur.2006.11.062

[28] Kim HK , Lee YJ , Han KN , Choi YH . Pulmonary function changes over 1 year after lobectomy in lung cancer. Respir Care. 2016;61:376–82. 26604331 10.4187/respcare.04284

[29] Pelletier C , Lapointe L , LeBlanc P . Effects of lung resection on pulmonary function and exercise capacity. Thorax. 1990;45:497–502. 2396230 10.1136/thx.45.7.497 PMC462576

[30] Nezu K , Kushibe K , Tojo T , Takahama M , Kitamura S . Recovery and limitation of exercise capacity after lung resection for lung cancer. Chest. 1998;113:1511–6. 9631786 10.1378/chest.113.6.1511

[31] Wei S , Chen F , Liu R , Fu D , Wang Y , Zhang B , et al. Outcomes of lobectomy on pulmonary function for early stage non‐small cell lung cancer (NSCLC) patients with chronic obstructive pulmonary disease (COPD). Thorac Cancer. 2020;11:1784–9. 32374491 10.1111/1759-7714.13445 PMC7592038

[32] Long J , Petrov R , Haithcock B , Chambers D , Belanger A , Burks AC , et al. Electromagnetic transthoracic nodule localization for minimally invasive pulmonary resection. Ann Thorac Surg. 2019;108:1528–34. 31233723 10.1016/j.athoracsur.2019.04.107

[33] Kreppel M , Zöller J . Ameloblastoma‐clinical, radiological, and therapeutic findings. Oral Dis. 2018;24:63–6. 29480593 10.1111/odi.12702

[34] Gilijamse M , Leemans CR , Winters HA , Schulten EA , van der Waal I . Metastasizing ameloblastoma. Int J Oral Maxillofac Surg. 2007;36:462–4. 17275258 10.1016/j.ijom.2006.12.005

[35] Laughlin EH . Metastasizing ameloblastoma. Cancer. 1989;64:776–80. 2663133 10.1002/1097-0142(19890801)64:3<776::aid-cncr2820640335>3.0.co;2-8

